# High infectivity and waterborne transmission of seagrass wasting disease

**DOI:** 10.1098/rsos.240663

**Published:** 2024-08-07

**Authors:** Morgan E. Eisenlord, M. Victoria Agnew, Miranda Winningham, Olivia J. Lobo, Alex D. Vompe, Bryanda Wippel, Carolyn S. Friedman, C. Drew Harvell, Colleen A. Burge

**Affiliations:** ^1^ Department of Ecology and Evolutionary Biology, Cornell University, Ithaca, NY 14853, USA; ^2^ Institute of Marine Environmental Technology, University of Maryland Baltimore County, Baltimore, MD 21202, USA; ^3^ Department of Microbiology, Oregon State University, Corvallis, OR 97331, USA; ^4^ School of Aquatic and Fishery Sciences, University of Washington, Seattle, WA 98195, USA; ^5^ Department of Microbiology and Immunology, University of Maryland Baltimore, Baltimore, MD 21201, USA; ^6^ California Department of Fish & Wildlife, University of California, Davis Bodega Marine Laboratory, Bodega Bay, CA 94923, USA

**Keywords:** seagrass wasting disease, transmission, plant–pathogen interactions, marine disease, *Zostera marina*, *Labyrinthula zosterae*

## Abstract

Pathogen transmission pathways are fundamental to understanding the epidemiology of infectious diseases yet are challenging to estimate in nature, particularly in the ocean. Seagrass wasting disease (SWD), caused by *Labyrinthula zosterae*, impacts seagrass beds worldwide and is thought to be a contributing factor to declines; however, little is known about natural transmission of SWD. In this study, we used field and laboratory experiments to test SWD transmission pathways and temperature sensitivity. To test transmission modes in nature, we conducted three field experiments out-planting sentinel *Zostera marina* shoots within and adjacent to natural *Z. marina* beds (20 ± 5 and 110 ± 5 m from bed edge). Infection rates and severity did not differ among outplant locations, implicating waterborne transmission. The infectious dose of *L. zosterae* through waterborne exposure was assessed in a controlled laboratory experiment. The dose to 50% disease was 6 cells ml^−1^ and did not differ with the temperatures tested (7.5°C and 15°C). Our results show *L. zosterae* is transmissible through water without direct contact with infected plants. Understanding the transmission dynamics of this disease in the context of changing ocean conditions will improve *Z. marina* protection and restoration in critical coastal habitats worldwide.

## 1. Introduction

Transmission rates are often the most sensitive parameter in theoretical host–parasite models and yet are extremely challenging to estimate in nature and particularly in the ocean [1,2]. Pathogen transmission may be fundamentally different in aquatic and marine habitats from land because saltwater is a favourable medium that allows dispersive stages to remain free-living outside their hosts for extended periods and travel long distances to infect susceptible populations [3]. Thus, many critical epidemiological parameters that are needed to predict outbreaks and effects on population dynamics are still unknown for the majority of marine pathogens, even as disease outbreaks are projected to increase in warming oceans [4–6].

Diseases on land can transmit through vertical or horizontal (direct and/or indirect) transfer within a population [7]. How diseases are transmitted within and among populations can greatly influence their impact on host populations both locally and regionally. In the ocean, a majority of pathogens have multiple transmission modes, and each mode can include complex routes to infect susceptible hosts such as direct contact, indirect contact through the water column or through vectors [8]. The relative contribution of direct versus indirect host contacts in the infection process influences the epidemiology of infectious diseases and impacts the ability to predict disease outbreaks [9]. Global climate change is increasing disease risk in many marine species [6,10] and is shifting infectious disease patterns in the ocean, leading to increased pathogen transmission rates, pathogenicity (disease severity in infected hosts) and greater host susceptibility to infection [11]. Therefore, determining the relative importance of possible transmission modes within specific host–pathogen systems and diverse environments is vital to a realistic understanding of disease dynamics [7], especially in changing climates.

Seagrass meadows are an essential marine habitat, endangered by a range of threats worldwide [12], including an infectious wasting disease affecting temperate seagrasses. As the only true flowering marine plant, seagrass plays an especially vital role in the nearshore ecosystem. Risk of pathogenic disease outbreaks in plants is increasing owing to climate change through shifts in host–pathogen interactions, increased spread and range shifts facilitated by climate [13]. These outbreaks lead to socio-economic and ecological harm. *Zostera marina*, the dominant habitat-forming seagrass species in temperate ecosystems along US Atlantic and Pacific coasts, is in decline [14–18], partially from seagrass wasting disease (SWD). During the 1930s an outbreak of SWD on the Atlantic coasts of Europe and the USA caused *Z. marina* losses of up to 90% with widespread ecological impacts that disrupted coastal food chains and sedimentary processes [19,20]. Following this, smaller outbreaks of SWD have been documented in many locations globally [21]; however, the disease has not been systematically studied across *Z. marina’s* growing range. Despite the threat SWD poses to the stability of *Z. marina* and other seagrass species, there is currently not a clear enough understanding of the epidemiology of the disease to build theoretical models for its impacts under future climate conditions.

Evidence from field studies suggests that SWD in *Z. marina* is widespread and outbreaks may be facilitated by environmental stress, but prevalence and severity are highly variable between beds and geographic regions [16,17,20–24]. However, mechanisms of transmission have not been studied in nature, limiting the ability to develop predictive epidemiology models, the accuracy of which (particularly in plants) depends on an understanding of transmission dynamics [25,26]. SWD is characterized by black or brown lesions on the leaf tissue, often with a pale centre, that are associated with pathological signs such as necrosis and loss of photosynthetic capabilities owing to deterioration of chloroplasts [27,28]. *Labyrinthula zosterae* was confirmed as the aetiological agent of SWD in the 1980s using Koch’s postulates with direct leaf-to-leaf contact [27]. Owing to these foundational studies using leaf-to-leaf contact, it has long been considered the dominant transmission mode in nature despite this hypothesis not having been experimentally tested. Controlled experiments on environmental factors impacting SWD severity post-infection have used both direct leaf contact and waterborne transmission as exposure methods, suggesting that both are effective while not explicitly testing transmission mode in nature or pathogen dose [23,29,30]. Other forms of transmission, i.e. through the roots or with vectors are possible but remain understudied; recent studies suggest that bivalves may aid in the transmission of *L. zosterae* but the mechanism is unknown [31,32]. It has been suggested that waterborne transmission may play an important role in the spread of this pathogen [32,33], and that a better understanding of modes of transmission could lead to mitigation and preventative strategies in the future [31]. Collectively, studies indicate warming temperatures may increase the severity of SWD, but do not address the effect of temperature on transmission [17,30,34–36]. The lack of experiments on SWD transmission dynamics leaves critical gaps in our knowledge of the basic biology of the host–pathogen interaction and limits our ability to assess risk and predict future outbreaks [32].

In this study, we quantified SWD transmission in both field and laboratory experiments. To test *in situ* transmission modes, we out-planted sentinel *Z. marina* shoots in three field experiments over consecutive years. Shoots were suspended in the water column within the natural *Z. marina* beds and 20 ± 5 m outside the *Z. marina* beds to test near-bed transmission. In the third experiment, shoots were also out-planted at 110 ± 5 m from the *Z. marina* bed to test the effect of distance. In a controlled laboratory experiment, we assessed the *L. zosterae* infectious dose to 50% (ID_50_) and the effect of varying temperatures on SWD prevalence and severity. Through field and laboratory experiments we tested three hypotheses: (i) in the natural environment, transmission of *L. zosterae* can occur without direct contact through transport in the water column, and low doses in the water can infect new host individuals; (ii) increased ocean temperature will increase *L. zosterae* prevalence and SWD lesion severity; and (iii) *Z. marina* growth rates will be lower in shoots infected with *L. zosterae* and with more severe infections. This is the first study to examine SWD transmission in the field and to calculate the *L. zosterae* infectious dose of waterborne *L. zosterae* transmission.

## 2. Material and methods

### 2.1. Sentinel *Zostera marina* field experiments

#### 2.1.1. Sentinel experiment design

We developed an *in situ* method to quantify SWD transmission in field experiments over three consecutive summers (2017, 2018 and 2019). Experiments were conducted between June and July of each year when SWD is prevalent in regional *Z. marina* beds [16,37]. Study sites were selected from a group of *Z. marina* beds surveyed for endemic SWD between 2013 and 2021 [24]. Selection criteria included presence of a persistent *Z. marina* bed, bathymetry of the site and consistent recorded presence of SWD. Previous sampling detected amplifiable *L. zosterae* DNA, a proxy for pathogen presence [31], in lesioned tissue identified as SWD at all three study sites (M Eisenlord 2024, unpublished data).

The sentinel method was used at the following three field sites in the San Juan Islands, WA, USA: False Bay Marine Preserve (48.482864, −123.073923), San Juan Island (2017); Beach Haven (48.694077, −122.948633), Orcas Island (2018); and Fourth of July Beach (48.463713 N, −122.991055 W), San Juan Island (2019). These studies tested the hypotheses that transmission of *L. zosterae* can occur in nature through the water column as well as through contact with infected *Z. marina* plants (table 1).

**Table 1.** Summary of location, timing and replication across treatments for the three sentinel *Z. marina* experiments run in 2017, 2018 and 2019.

years	source population	deployment location	deployment date	# units inside *Z. marina* bed	# units near *Z. marina* bed	# units distant to *Z. marina* bed	# shoots/unit	total # shoots deployed	# lab controls	# leaf samples for qPCR
2017	Fourth of July Beach	False Bay	29/6 to 19/7	6	6	NA	5	60	30	40
2018	Beach Haven	Beach Haven	13/6 to 28/6	6	6	NA	6	72	36	17
2019	Fourth of July Beach	Fourth of July Beach	19/6 to 3/7	8	8	8	6	180	36	27

For each study, *Z. marina* shoots were haphazardly collected 3 days prior to the planned outplant approximately 2 m from one another at a depth of approximately 1.5 feet below Mean Lower Low Water. After collection, shoots were immediately transported to Friday Harbor Labs, WA (FHL). Owing to SWD being endemic at collection sites, shoots usually showed signs of disease at the time of collection in the oldest leaves. Upon arrival at FHL, shoots were cleaned of epiphytes and standardized to include only the youngest two leaves free of SWD signs. Leaves were cut to 15 cm in length and rhizomes were cut to three nodes. To minimize post-collection stress, all experimental and control shoots were then held in chambers containing 30 ppt 1 µm filtered seawater (FSW) for 3 days at ambient temperature. Prior to the experiment, shoots were placed in a 20 ppt low salinity treatment overnight to reduce the presence of surface *L. zosterae* cells (1 µm FSW and reverse osmosis fresh water). Following this, a pin prick was made through all leaves at the top of the sheath to allow visual separation of new growth from pre-existing tissue, and shoots were rinsed in reverse osmosis fresh water then either transported submerged in FSW to the field site or placed into control tanks that remained at FHL.

**Figure 1.**
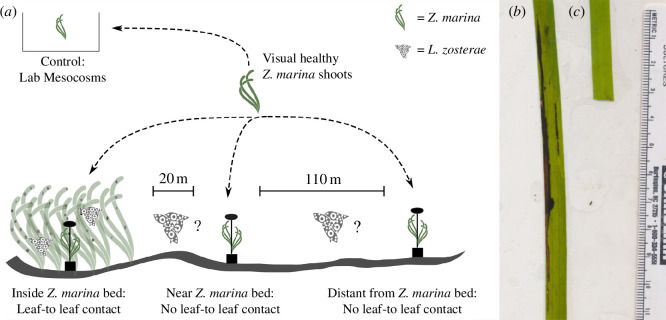
Sentinel *Z. marina* natural *L. zosterae* transmission experiment design. (*a*) Standardized *Z. marina* shoots visually free of SWD were divided into four treatments: inside the *Z. marina* bed (all 3 years), approximately 20 ± 5 m from the edge of the *Z. marina* bed (all 3 years) and approximately 110 ± 5 m from edge of the *Z. marina* bed (2019 only). Sentinel *Z. marina* shoots were attached to each experimental unit. The control shoots were divided into replicate tanks and held in the lab under controlled conditions for the duration of the experiment. (*b*) Example of SWD positive *Z. marina* leaf showing signs of *L. zosterae* infection. (*c*) Example of SWD negative *Z. marina* leaf.

Sentinel *Z. marina* shoots were deployed in the field attached to experimental units that each consisted of a cinderblock, rope and Styrofoam ‘float’ that allowed the unit to hold 5–6 shoots vertically in the water column (figure 1). In all three sentinel studies experimental units were deployed inside the *Z. marina* bed (inside treatment location) and 20 ± 5 m outside the *Z. marina* bed edge (near treatment location) for 14 days at a depth of approximately 1.5 feet below Mean Lower Low Water. In 2019, an additional distance of 110 ± 5 m outside the *Z. marina* bed edge was added (distant treatment location). See table 1 for details on the site, number of replicates and timing of each experiment. Laboratory controls were divided into six tanks, each holding approximately 12 l FSW and maintained in a temperature-controlled room with aeration and lights (diel 16 : 8 light : dark cycle) in 0.22 µm FSW. On the last day of the experiment, shoots were collected from the field, placed in 1 µm FSW and transported to FHL for processing.

#### 2.1.2. *Zostera marina* tissue processing

Leaves were cut at the top of the sheath, carefully cleaned of epiphytes and debris, placed between two sterile, clear plastic sheets and imaged using a Canon CanoScan 9000F Mark II scanner at 600 dpi. We used visual signs of SWD as a proxy for *L. zosterae* infection [31,38]. All of the deployed shoots were characterized for SWD prevalence (per cent of shoots with visual signs of SWD) and SWD severity (per cent of blades covered by SWD lesions), and total growth of each leaf in ImageJ [39] using scanned images of the leaves.

To confirm the presence of *L. zosterae* DNA within diseased leaves, and the absence of *L. zosterae* DNA in healthy leaves, a subset of 17–40 leaves each year were haphazardly selected from each treatment loaction and categorized as positive or negative for SWD based on visual signs (table 1). Each of these leaves was placed in sterile seawater and vortexed for 30 s on high to remove *L. zosterae* remaining on the surface of the leaf. For quantitative polymerase chain reaction (qPCR) confirmation of *L. zosterae* involvement, one 1.5–2 cm^2^ lesion was aseptically excised from each visually positive leaf. A similarly sized sample of tissue with no lesions was selected from each negative leaf. The excised section was rinsed with reverse osmosis water, flash-frozen in liquid nitrogen and stored at −80°C until DNA extraction.

The qPCR was used as a proxy for infection to detect and quantify *L. zosterae* DNA abundance in diseased and visually healthy leaf tissue in samples taken from both the sentinel and ID_50_ experiments. Frozen and lyophilized *Z. marina* leaf tissue (approx. 60 mg) was ground to a fine powder using a Qiagen TissueLyser II (25 s^−1^ speed for 5 min). Following the methods outlined in Groner *et al*. [23], total DNA was extracted from the *Z. marina* tissue using the Qiagen DNeasy Plant Mini Kit.

Copies of *L. zosterae* DNA were quantified using qPCR methods from Bockelmann *et al*. [40] as modified by Groner *et al*. [23] and Agnew *et al*. [31]. The qPCR assay targeted the internal transcribed spacer (ITS) region using the forward (Laby_ITS_Taq_f: 5′−TTG AAC GTA ACA TTC GAC TTT CGT−3′) and reverse (Laby_ITS_Taq_r: 5′–ACG CAT GAA GCG GTC TTC TT−3′) primers, and TaqMan probe (Laby_ITS_probe: 5′−FAM−TGG ACG AGT GTG TTT TG –MGB-NFQ−3′). Each 20 μl reaction included 10 μl of TaqMan Fast Universal PCR Master Mix (Applied Biosystems by Life Technologies), 400 nm of each primer, 15 μg of BSA, 100 nm of probe and 2 µl of DNA that was previously diluted 1 : 10. The plasmid standard curve ranged from 3 × 10^7^ to 3 copies of *L. zosterae* DNA [31]. The standard curve and samples were run in duplicate in 96-well microplates using an Applied Biosystems 7500 Fast Real-Time PCR System with the following reaction conditions: 95°C for 20 s, followed by 40 cycles of 95°C for 3 s and 60°C for 30 s. The reaction efficiency ranged between 101.2 and 102.9% and *R* ^2^ > 0.99 for the standard curve.

### 2.2. ID_50_ laboratory experiment

#### 2.2.1. Experimental design

In the laboratory-based ID_50_ challenge experiment, we explored waterborne *L. zosterae* transmission under controlled conditions. The experiment was conducted at the FHL Ocean Acidification Experimental Laboratory following protocols required by the state of Washington for working with *L. zosterae* in these facilities. The experiment used an ID_50_ experimental design testing both *L. zosterae* dose and temperature.

We conducted the experiment in May 2019, to collect newly emerged *Z. marina* shoots with minimal prior exposure to SWD (M Eisenlord 2024, unpublished data); however, owing to SWD being endemic at collection sites, some shoots inevitably showed signs of disease at the time of collection on the oldest leaves. Shoots were haphazardly collected from Fourth of July Beach on 9 May 2019, and transported to FHL. After collection, shoots were cleaned, trimmed and processed as in §2.1.1. Shoots were then placed into static chambers (*n* = five shoots/chamber) in 3.5 l of 0.22 µm FSW. The chambers were placed into four recirculating water baths held at 10°C (ambient seawater temperature at the collection site) under full spectrum LED lights on an 16 : 8 light : dark cycle to reduce stress prior to inoculation. The following morning, shoots were standardized to include only the two youngest blades with no visual signs of SWD cut to 15 cm in length. Roots were also trimmed to include three nodes with roots, and plants were left to acclimatize overnight at their appropriate experimental temperatures (7.5 or 15°C). Subsequently, shoots were placed into individual 18-oz Whirl-Pak bags filled with FSW and inoculated.

The *L. zosterae* inoculum was prepared as follows. An isolate of *L. zosterae* originating from the seagrass collection site (Fourth of July Beach), the same isolate used in Agnew *et al*. [31] and previously determined to be virulent was used for the ID_50_ laboratory experiment. The *L. zosterae* isolate was grown on serum seawater agar (SSA) plates containing antibiotics as Porter [41] and modified by Groner *et al*. [23] at room temperature for 4 days. The resulting cells were scraped from the plates and suspended in 0.22 µm FSW. Zirconia/silica beads (1 µm) were added to the cells prior to being vortexed to break up ectoplasmic nets between cells and prevent clumping [35]. Subsequently, cells were enumerated using a haemocytometer and diluted to produce an initial stock with a concentration of approximately 1 × 10^6^ cells ml^−1^. The initial stock was serially diluted (10-fold) five times in 0.22 µm FSW to create six stock concentrations ranging from 1 × 10^2^ to 1 × 10^6^ *L. zosterae* cells ml^−1^. The 1 × 10^3^ concentration was diluted to 0.5 × 10^3^ to add an additional low-dose treatment falling between 1 and 10 *L*. *zosterae* cells ml^−1^. A sham control consisted of FSW only. Then 3.5 ml of stock inoculum at the assigned concentration was added to each WhirlPack (containing 346.5 ml of 0.22 µm FSW and one *Z. marina* shoot) to produce the resulting seven treatment doses of *L. zosterae* in a total of 350 ml of FSW: 10^4^, 10^3^, 10^2^, 10, 5, 1 and 0 (control) *L. zosterae* cells ml^−1^.

Three replicate 1 ml samples were taken from each inoculum and immediately frozen at −80°C for qPCR analysis of *L. zosterae* DNA concentration. Each dose was replicated eight times at both 7.5 and 15°C, for a total of 112 shoots (56 shoots per temperature). Plants were exposed to each *L. zosterae* dose for 24 hours before being placed into a new Whirl-Pak bag containing 350 ml of 0.22 µm FSW. See electronic supplementary material, figure S1 for diagram of ID_50_ experimental design.

Whirl-Pak bags were randomly distributed among seven 3.5 l static tanks that were held within larger recirculating freshwater baths at the appropriate temperature to reduce the risk of contamination. Each 3.5 l water bath contained four *Z. marina* shoots in individual bags with a randomly assigned *L. zosterae* dose so that each tank contained sealed Whirl-Pak bags with multiple treatment doses. The shoots were checked every 12 hours for signs of disease. The disease was identified as a black lesion on the *Z. marina* blade. Positive and negative disease signs were recorded until the end of the experiment, which ran for 132 hours.

#### 2.2.2. *Zostera marina* tissue processing

*Zostera marina* shoots were scanned as described in §2.1.2. All visual analysis of SWD prevalence was conducted blind to the DNA analysis. Following scanning, a 3 cm section was cut from each leaf using a sterile technique and placed directly into a nuclease and DNA/RNA-free clean 1.5 ml Eppendorf tube. To verify that lesions were a result of *L. zosterae*, the 3 cm section always included the outer edge of the largest lesion present, or the top 3 cm of the shoot if no lesions were present. Two samples were taken per shoot, one section from each of the two oldest leaves that were originally exposed to *L. zosterae*. These sections were immediately frozen at −20°C and moved to −80°C until DNA extraction. For each shoot, the sample with the largest lesion as determined by ImageJ analysis was chosen for qPCR analysis. DNA extractions and qPCR analysis were conducted following the same methods as the sentinel experiments (outlined in §2.1.2). To ensure the inoculums used for the ID_50_ experiment contained *L. zosterae* DNA, we collected three 1 ml subsamples from each dose directly after serial dilution and before adding to *Z. marina* treatments. Samples were centrifuged for 30 s at 2500 r.p.m. to concentrate the cells and 800 μl of liquid was pipetted off, leaving a volume of 200 μl for DNA extraction. Total DNA was extracted from the concentrated samples using the Qiagen DNeasy Blood & Tissue Mini Kit.

### 2.3. Statistical analysis

Data exploration was conducted preceding hypothesis testing following a structured method [42]. All analyses were run in the software package R v. 4.0.2 [43]. An alpha of 0.05 was set for all significance tests. In all cases, we constructed the most complete (maximal) generalized linear mixed model (GLMM) using a hypothesis testing methodology and compared submodels and interaction effects as appropriate for the dataset using corrected Akaike information criterion (AICc) (‘bbmle’; [44]). All models with ΔAICc < 3 from the best-fit model were reported. Unless otherwise noted, all models selected were the best-fit model. The R package ‘glmmTMB’ was used for fitting GLMMs as it provided flexibility in the model specification and the capacity to model zero-inflated data with a number of prior distributions (‘glmmTMB’; [45]). Random effects with less then five levels were not included in the models [46]. Zero-inflation models were selected for SWD severity, as sampling zeros as well as structural zeros can occur in this dataset since both visual and DNA identification of SWD may include infections under the detection threshold. GLMM *p*-values were calculated using likelihood ratio tests of the null versus full models (‘anova’). Coefficient confidence intervals and *p*-values reported for the GLMMs are Wald estimates. To investigate differences between treatment groups in the fitted GLMMs, multiple means were compared with Tukey contrasts (‘emmeans’; ‘multcomp’; [47,48]). Figures were generated using the R packages (‘ggplot2’ and ‘survminer’ [49,50]. Models selected met diagnostic criteria for GLMMs unless otherwise indicated (‘DHARMa’; [51]).

#### 2.3.1. Sentinel *Zostera marina* field experiments

We used GLMM models to analyse SWD prevalence (per cent of plants visually positive for SWD lesions) and severity (per cent of blade area with SWD lesions) in all three sentinel *Z. marina* experiments with *treatment* (inside versus near *Z. marina* bed) and *year* as fixed factors. Prevalence, severity and leaf growth in the 2019 experiment were modelled separately to test the effect of the additional sentinel distance outside the *Z. marina* bed (near = 20 ± 5 m and distant = 110 ± 5 m) with *treatment* as a fixed factor. To investigate the difference in SWD prevalence we ran binomial GLMMs with a log link function. SWD severity was modelled using zero-inflated beta GLMMs with a log link function. *Zostera marina* shoot growth h^−1^ was modelled with *treatment* and *SWD severity* as fixed factors using a GLMM under a Gaussian distribution. A random intercept for *block* was included for all models.

#### 2.3.2. ID_50_ laboratory experiment

The *L. zosterae* infectious dose to 50% (ID_50_) for *Z. marina* was calculated at the end (132 hours post-exposure) of the temperature–dose challenge experiment. Separate ID_50_ calculations were done for SWD presence/absence using visual disease signs and *L. zosterae* DNA measures; however, visual disease signs were considered the more relevant measure as they show host response. We calculated the ID_50_ value for each temperature and the overall ID_50_ using a generalized linear model (GLM) with a binomial distribution using the ‘dose.p’ function [52–54]. We selected this method as it allowed us to calculate standard errors and confidence intervals around the observed infection values, which is not possible using the Reed–Muench (RM) method of calculating ID_50_ values [55,56]. The ID_50_ and value were calculated using the serial dilution values for the target cell counts at each challenge dose. While the log_10_(*x* + 1) transformed dose provided the lowest standard error, these error values represented were approximately 0.1–0.2 cells. Error values of less than a cell does not represent biologically feasible proportions. Based on this and a model selection methodology of avoiding transformations unless necessary, we selected the model using the untransformed data for all statistical comparisons and rounded to the nearest whole cell. We tested for significant differences between the calculated ID_50_ value for each temperature using the Welch–Satterthwaite (W-S) two-tailed *t*‐test correction (‘stats’; [43]).

The effect of *L. zosterae* dose and temperature on the risk of infection after exposure and the rate at which *L. zosterae*-exposed shoots developed SWD signs were analysed using a Cox proportional hazards (Coxph) model (‘survival’; [57]). The model included fixed covariates for *temperature*, *dose* and the interaction between *temperature* and *dose*. SWD severity was modelled with a zero-inflated beta GLMM with a log link function and fixed covariates for *temperature* and *dose*. We modelled mean *Z. marina* leaf growth h^−1^ using a GLMM with a Gaussian distribution as a function of *temperature* and *SWD severity*. A random intercept for *tank* was included in the severity and growth models to account for the blocked experiment design.

Differences in mean *L. zosterae* DNA copies between the inoculum stock doses were compared using analysis of variance (ANOVA). To understand the degree of agreement between visual infection signs and DNA measures of *L. zosterae* presence/absence we ran an intraclass correlation (ICC) analysis for two raters (‘irr’; [58]). We calculated the ICC on the full dataset and the dataset subset by SWD lesion area less then 0.5 mm^2^ to identify where the greatest discrepancies between the visual (number of plants with visual SWD lesions) and DNA (number of plants containing *L. zosterae* DNA) disease prevalence occurred.

## 3. Results

### 3.1. Sentinel *Zostera marina* field experiments

Mean SWD prevalence (% ± s.e.) was 8 ± 4 for the shoots held in control tanks, 79 ± 9 for the inside bed treatment with leaf-to-leaf contact, 84 ± 5 for the near bed treatment (20 ± 5 m) and 67 ± 7 for the distant treatment (110 ± 5 m, 2019 only). Median severity (% total blade area with SWD lesions ± interquartile range (IQR)) of shoots positive for SWD was 1.3 ± 1.7 in the control, 1.3 ± 4.3 inside the bed, 1.8 ± 6.8 near the bed and 2.3 ± 7.0 distant from the bed (2019 only). *Zostera marina* shoots exhibited positive leaf growth (mean growth mm^2^ h^−1^ ± s.e.) in all treatments (control = 9 ± 0.2, inside = 8 ± 0.4, near = 6 ± 0.3, distant = 5.7 ± 0.3). Tissue samples visually categorized as negative for SWD signs contained no amplifiable *L. zosterae* DNA. The presence of *L. zosterae* DNA was confirmed by qPCR in the subset of tissue samples from visually positive SWD shoots in each treatment over all three sentinel field experiment years. Sample tissue weights were measured in all years but are only available for 2019. Mean *L. zosterae* DNA (copies mg^−1^ dry *Z. marina* ± s.e.) in visually positive SWD shoots by treatment location were 1.0 × 10^6^ ± 4.5 × 10^5^ inside the bed, 1.7 × 10^5^ ± 8.2 × 10^4^ near the bed and 8.1 × 10^5^ ± 6.0 × 10^5^ distant from the bed. Since *Z. marina* tissue samples taken for qPCR were of equivalent size in all years, we also calculated mean *L. zosterae* DNA for the inside and near bed treatments overall. Mean *L. zosterae* DNA (copies ± s.e.) were 5.2 × 10^6^ ± 1.0 × 10^6^ inside the bed and 1.8 × 10^6^ ± 7.8 × 10^5^ near the bed. See electronic supplementary material, table S3 for data summary of sentinel experiments.

**Figure 2.**
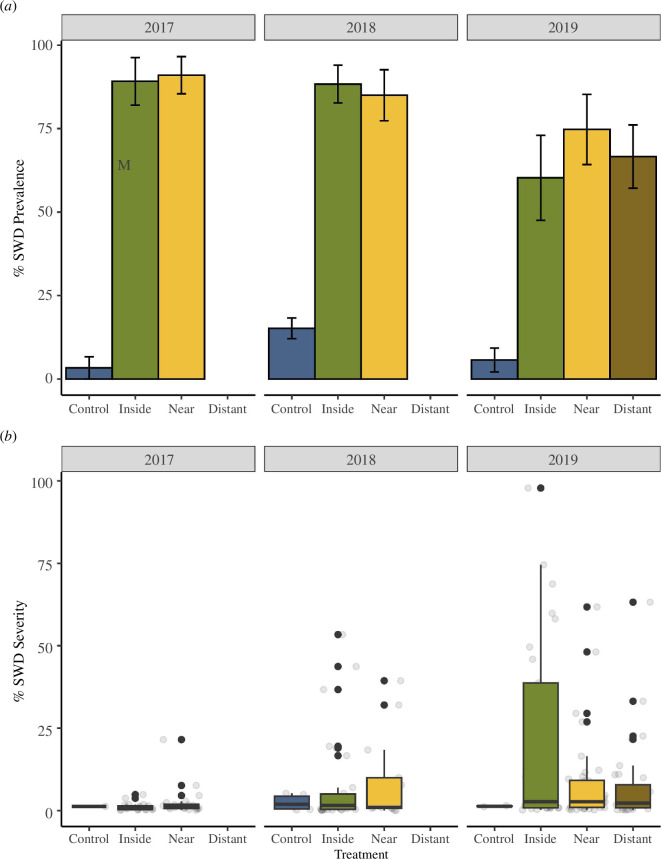
Sentinel *Z. marina* field experiments 2017–2019. (*a*) SWD prevalence in the sentinel *Z. marina* shoots measured as the number of shoots with visual SWD signs. Data is shown as the mean % positive shoots. (*b*) SWD severity in the sentinel *Z. marina* shoots. Data are shown as box and whisker plots depicting median (line within box), IQR (box) and full data spread (grey dot = sample; black dot = sample outlier from IQR) of all *Z. marina* shoots with positive signs of disease.

Risk of SWD infection in the sentinel *Z. marina* shoots was impacted by treatment location (inside, near, or distant) in all years (GLMM, marginal *R* ^2^ = 0.536, conditional *R* ^2^ = 0.642; *p* < 0.001, figure 2*a*). Pairwise comparisons showed an equally increased risk of SWD in all sentinel *Z. marina* shoots placed inside or near the *Z. marina* bed over those kept in the laboratory control tanks in all years. The severity of SWD in infected shoots did not vary by the treatment location or year/site (GLMM, marginal *R* ^2^ = 0.060; *p* = 0.1849; figure 2*b*). See electronic supplementary material, table S1 for model outputs.

In the 2019 experiment, sentinel shoots deployed at distance (110 ± 5 m) from the bed had the same risk of SWD infection as the inside bed and near bed treatments (GLMM, marginal *R* ^2^ = 0.40, conditional *R* ^2^ = 0.60; *p* < 0.001). SWD severity of infected shoots did not differ between the inside, near and distant treatments (GLMM, marginal *R* ^2^ = 0.04, *p* = 0.64). There was no difference in growth between the inside, near or distant treatments; however, the control treatment showed increased growth as expected (GLMM, marginal *R* ^2^ = 0.18, *p* < 0.001). Higher SWD severity was negatively correlated with *Z. marina* growth rates (*p* < 0.001) and accounted for 53% of the variation in shoot growth rates. See electronic supplementary material, table S2 for model outputs.

Mean water temperatures at all three sites during 2017–2018 can be found in Graham *et al*. [59]. Mean water temperature during the 14 days 2019 deployment at Fourth of July Beach was (mean ± s.d.) 11.8 ± 0.7°C inside the *Z. marina* bed, 11.6 ± 0.5°C near the *Z. marina* bed, 11.6 ± 0.5°C far from the bed and 13.7 ± 1.3°C in the laboratory control.

### 3.2. ID_50_ laboratory experiment

**Figure 3.**
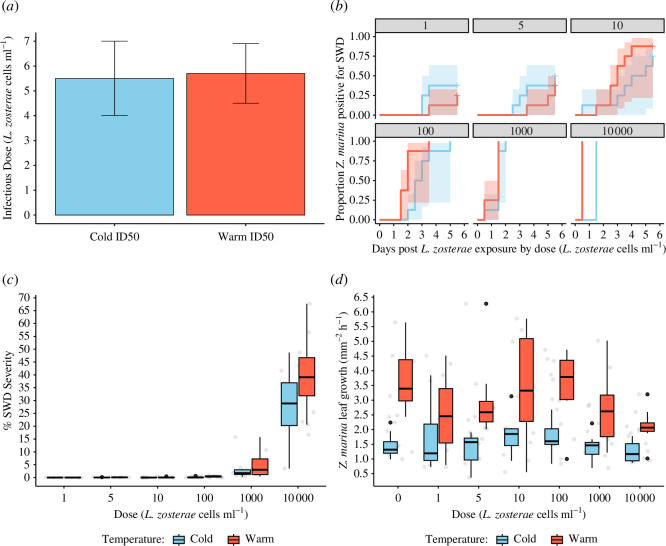
ID_50_ laboratory challenge experiment results. (*a*) The warm and cold ID_50_ cells ml^−1^ for each temperature (7.5 and 15°C). Error bars represent standard error. (*b*) Cumulative event Kaplan–Meier curves show the visual SWD signs development in *Z. marina* shoots from days 0 to 6. Red and blue shading represent confidence intervals calculated from the Kaplan–Meier curves. (*c*) %SWD severity in *Z. marina* shoots 6 days after exposure by dose and temperature. Data are shown as box and whisker plots depicting median (line within box), IQR (box) and full data spread (grey dot = sample; black dot = sample outlier from IQR) of all *Z. marina* shoots with positive signs of disease. (*d*) *Zostera marina* leaf growth rates h^−1^ by dose and temperature. Data are shown as box and whisker plots depicting median, IQR (box) and full data spread from all *Z. marina* shoots. The data are shown by dose, with the 7.5°C (blue) and 15°C (red) treatments compared.

The ID_50_ of *L. zosterae* using visual signs of disease was calculated as 6 ± 1 s.e. *L. zosterae* cells ml^−1^ (figure 3*a*). The ID_50_ values did not differ between the cold and hot temperatures (*p* = 0.74). Using the number of *L. zosterae* DNA positive samples to calculate the ID_50_ produced value of 7 ± 1 s.e. *L. zosterae* cells ml^−1^. The ID_50_ values calculated using positive visual disease signs of SWD versus *L. zosterae* DNA positive samples were not significantly different.

*Labyrinthula zosterae* dose greater than 10^2^ *L. zosterae* cells ml^−1^ increased the hazard ratio of developing SWD (Coxph, Wold test = 99.4, *p* < 0.001; figure 3*b*). The hazard of developing SWD did not differ between shoots exposed to 1, 5 and 10 *L. zosterae* cells ml^−1^. The hazard of developing SWD for shoots exposed to 10^2^ *L. zosterae* cells ml^−1^ was approximately 8× greater than exposure of 1 *L. zosterae* cell ml^−1^ (*p* = 0.003). The hazard of developing SWD at doses of 10^3^ and 10^4^ *L. zosterae* cells ml^−1^, respectively, was approximately 106× and approximately 130× greater than exposure to 1 cell ml^−1^ (*p* < 0.001). The temperature did not affect the hazard ratio of developing SWD (*p* = 0.5); however, at the 10^4^ *L. zosterae* cells dose warm temperature increased the hazard ratio for developing SWD compared with the cold temperature (*p* = 0.004). SWD severity increased with higher *L. zosterae* exposure dose and temperature (GLMM, marginal *R* ^2^ = 0.898, conditional R^2^ = 0.960, *p* < 0.001; figure 3*c*). Pairwise comparisons showed severity was significantly higher in the 1000 (approx. 29×) and 10 000 (approx. 300×) *L. zosterae* cells ml^−1^ exposure doses (*p* < 0.001) than in the 1, 5, 10 and 100 *L. zosterae* cells ml^−1^, which did not differ from each other in severity. The severity increased slightly in the warm temperature (*p* = 0.014). *Zostera marina* growth rates were doubled in the 15°C versus 7.5°C treatment and decreased by higher SWD severity (GLMM, marginal *R* ^2^ = 0.368, conditional *R* ^2^ = 0.402, *p* < 0.001; figure 3*d*). See electronic supplementary material, table S4 for model outputs.

A strong positive correlation was observed between *L. zosterae* DNA copies and SWD severity in the tissue sections (Spearman rank correlation, *R* ^2^ = 0.91; *p*‐value < 0.001; electronic supplementary material, figure S2). However, not all visual and DNA presence/absence agreed. Samples where the visual and DNA measures of assessing SWD prevalence did not agree had a higher rate of negatives using the *L. zosterae* DNA copies versus the visual signs as a method for evaluating disease prevalence, and most of those differences were in lesions less than or equal to 0.5 mm^2^. The overall ICC showed a 74% agreement between the visual and *L. zosterae* DNA prevalence measures (*p* < 0.001) but was reduced to 53% agreement in lesions less than 0.5 mm^2^ (*p* < 0.001).

All inoculum exposure doses contained amplifiable *L. zosterae* DNA at significantly different levels, while the negative control lacked any amplifiable *L. zosterae* DNA (ANOVA, *p*‐value < 0.001). Mean *L. zosterae* DNA copies ml^−1^ for inoculum stock doses before dilution into the treatment bags are as follows: 1 × 10^4^ = 6.6 × 10^7^ ± 6.3 × 10^6^, 1 × 10^3^ = 6.7 × 10^6^ ± 3.2 × 10^5^, 1 × 10^2^ = 2.4 × 10^5^ ± 3.1 × 10^4^, 10 × 10^1^ = 2.3 × 10^4^ ± 2.9 × 10^2^, 5 × 10^1^ = 5.8 × 10^3^ ± 7.5 × 10^2^ and 1 × 10^1^ = 2.4 × 10^3^ ± 4.8 × 10^2^. Mean temperatures during the experiment were (mean ± s.d.) 7.6 ± 0.2°C in the cold treatment and 14.7 ± 0.4°C in the warm treatment. See electronic supplementary material, table S5 for data summary of the ID_50_ experiment.

## 4. Discussion

This is the first study to demonstrate waterborne transmission of *L. zosterae* as an important mode of transmission in field trials. In the sentinel experiments, we showed that SWD prevalence and severity were not influenced by distance from *L. zosterae*-infected *Z. marina* beds. In addition, this is the first study to quantify the infectious dose of *L. zosterae* and demonstrate its infectivity—only 6 cells ml^−1^ were needed to infect a *Z. marina* shoot. Our results suggest SWD may spread rapidly and widely through the water column in natural marine habitats. Waterborne transmission of SWD may be a major contributor to infections in nature and an important factor to consider in the preservation of seagrass beds in *L. zosterae* endemic areas.

The demonstration of natural, in the field, waterborne transmission of *L. zosterae* and SWD in this study is novel. *Zostera marina* shoots deployed in this experiment were not caged, and therefore contact with diseased floating blades is possible, but probably not the sole method of transmission. The high level of infection in sentinel plants 100 m from a seagrass meadow suggests that waterborne transmission may play an equal or greater role than direct, contact-mediated transmission of SWD. Determining potential transmission modes for sessile marine species like *Z. marina,* which play essential roles in habitat formation and stabilization, is important because efficient transmission without direct contact greatly increases the risk of widespread impacts. Many marine diseases can be transmitted through the water column and this transmission mode plays an important role in the spread and maintenance of these diseases. Examples include many coral diseases such as those caused by the bacteria *Vibrio* spp., marine viruses *Panulirus argus* Virus 1 in spiny lobster (PaV1) and infectious haematopoietic necrosis virus in salmon (IHNV) [8,60,61]. The risk of extensive impacts is also affected by the virulence (high infectivity and pathogenicity) of a pathogen, and how the host–pathogen system is affected by environmental stressors. Transport of pathogens through ocean currents can facilitate widespread transmission and increased genetic diversity of pathogens. For example, a modelling study of PaV1 in the Caribbean found that the area with the highest genetic diversity of the pathogen also had the most ‘gateways’, or locations with high pathogen spread (usually through proximity to major oceanographic currents) [61]. Previous studies have shown multiple pathogenic *L. zosterae* strains occur within and among *Z. marina* beds, but it is not known to what degree the mixing of strains between *Z. marina* beds and regions occurs [29,35]. With waterborne transmission of SWD, widespread mixing of *L. zosterae* strains is likely, potentially benefitting the spread of newly evolving and highly successful *L. zosterae* strains.

Our results also highlight the necessity of establishing a visual limit of detection for SWD and standard diagnostics. The use of qPCR diagnostic assays for pathogens such as withering syndrome in abalone and parasites in oysters usually results in increased positive results compared with visual diagnostics such as histopathology [62–64]. In this study, we found a strong correlation between SWD severity (visual) and *L. zosterae* DNA copies (molecular), consistent with other studies on SWD which compare both approaches as diagnostic methods [31,65]. While this held true for the majority of samples, some small black spots less than 0.5 mm^2^ positively identified as SWD signs, which show the black colour change suggestive of lesions but are too small to identify other characteristics of SWD lesions, were more likely to be negative for *L. zosterae* DNA in the qPCR assay than larger lesions. None of these black spots developed in the control shoots exposed to the sham inoculum. Based on these observations, we believe this mismatch is largely owing to *L. zosterae* infections below the limit of detection for our assay or visual signs of damage remaining after the plant has healed an unsuccessful infection attempt. Interestingly, another study on SWD found visual diagnostics overestimated the amount of *L. zosterae* DNA present in SWD lesions covering greater than 1/3 of the leaf area and suggest this may be owing to necrosis as a response to an old SWD infection and not active pathogen load [65]. Further investigation into the relationship between visual signs of SWD and *L. zosterae* pathogen load through the progression of the disease, combined with histopathology and microscopy to visualize the host–pathogen interaction, is needed to clarify this issue and develop standard SWD diagnostics.

Rapid transmission rates and reduced growth in SWD-infected shoots could impact *Z. marina* productivity over the course of a growing season. Our study demonstrated *L. zosterae* infections and development of SWD occurs quickly over two weeks, which is equivalent to the time it takes *Z. marina* plants to grow a new leaf [66]. In fact, by the end of the two weeks field exposure, SWD prevalence in the sentinel treatments had reached a similar level as in the natural *Z. marina* bed. *Zostera marina* leaf growth was reduced with increased SWD severity in the sentinel experiment, consistent with other studies showing decreased photosynthetic capacity and starch reserves [67] in seagrass with *L. zosterae* infection [34,68]. *Zostera marina* relies on energy reserves during the growing season to produce seeds and survive the winter [67]. By damaging photosynthetic cells, reducing growth and starch reserves, SWD can limit *Z. marina* resources during the critical growth period. This demonstrates the potential for both short- and long-term impacts of SWD on the health of wild *Z. marina* populations.

By measuring the risk of disease and severity separately, we were able to identify different drivers of the infection process. The dosage of a pathogen required to initiate disease is a vital component of transmission biology and a parameter needed to increase our understanding of the epidemiology of host–pathogen systems in nature. Our laboratory results suggest that increased temperature does not impact SWD severity or prevalence, except when combined with a high dose of *L. zosterae* exposure (10^4^ cells ml^−1^). Susceptible hosts show signs of disease after short exposures to 6 *L*. *zosterae* cells ml^−1^. The risk of SWD increased exponentially with exposure dose but was not significantly changed by temperature. These results are consistent with a dose–response pattern, with an exponential increase in risk as cell concentrations increase. There are multiple isolates and potential species of *Labyrinthula*, and the pathogenicity of virulent isolates can vary within a single host and between hosts [29,69]. Owing to this, the ID_50_ and response to temperature increases may vary depending on the isolate and host. Defining the ID_50_ of *L. zosterae* for a range of isolates is a necessary step towards disentangling infection processes and comparing infection rates to other host–pathogen systems.

Many host–pathogen relationships and resultant disease ranges and intensities are changing in response to warming oceans. Interestingly, we observed no influence of temperatures used in this study on disease rates for this isolate. Our ID_50_ experiment showed that *L. zosterae* causes disease with only a few cells ml^−1^ exposure over a wide range of temperatures, thus low water temperature may not protect shoots from initial infection. The treatment temperatures represented a large range covering the approximate summer maximum (15°C) and winter minimum (7.5°C) at the site where the *Z. marina* shoots and *L. zosterae* culture were collected. The lack of an effect of temperature except at the highest dose in the present study demonstrates that the ability of *L. zosterae* to successfully infect *Z. marina* is dependent on other factors. Environmentally relevant concentrations of *L. zosterae* cells needed to infect in nature are unknown, though results from the sentinel experiments suggest consistent levels over the infectious dose of 6 *L*. *zosterae* cells ml^−1^ occur in the field.

Minimal effects of increased temperature on *L. zosterae* infectivity have been seen in other studies. Short-term (5–8 days) increased temperature had no effect on lesion severity [30,34,35]; however, longer-term exposure (13–14 days) did result in increased lesion severity at higher temperatures [31,70]. Previous laboratory studies showed that warm temperatures (18°C) increased *L. zosterae* growth rates in culture compared with cooler temperatures (12°C) [35]. Importantly, compounding stressors in addition to temperature probably affect lesion severity. For example, some studies only detected significant increases in SWD severity with high temperatures when combined with low light conditions or high salinity [30,36]. These results show compounding factors affecting the hosts’ ability to fight off or negate the consequences of infection are important to consider in this host–pathogen system and are key in predicting the effect of increased temperature on SWD prevalence and severity.

Sentinel and controlled dose experiments provide repeatable methods for studying SWD transmission. Similar methods have been effectively used to study pathogens of marine decapods [71] and oysters [72,73], and in terrestrial systems to serve as early detection of invasive plant pathogens [74]. The sentinel *Z. marina* experiment design provides a framework for the assessment of SWD transmission dynamics and other host–pathogen interactions of seagrass *in situ*. We found consistent and repeatable results over three different years and field sites, showing that the experimental design is robust and can be used to compare SWD transmission across different locations, time frames and environmental conditions. Pairing sentinel studies in the field with controlled laboratory experiments, as we did in the 2019 experiments, enables quantification of metrics such as transmission rate coupled with measurements of virulence such as prevalence and severity of lesions, thus pinpointing mechanisms involved in the disease process. We incorporated multiple points of connection between laboratory and field experiments, for example, using *Z. marina* shoots and a virulent *L. zosterae* isolate cultured from the 2019 sentinel experiment site for the ID_50_ experiment. This design allows us to explore transmission dynamics in the natural system while controlling for potential local adaption in the host–pathogen interactions.

## 5. Conclusion

Infectious disease is an increasing threat to ecosystems worldwide and is projected to increase with rising temperatures and anthropogenic stress [6]. Mechanisms of infectious disease transmission are poorly understood in marine habitats yet are essential to predicting future disease outbreaks and identifying communities at particular risk. Unlike terrestrial habitats where pathogens must overcome the harsh environment of air to transmit between hosts, water provides an easier medium to transverse. Diseases impacting species like *Z. marina* that function as coastal ecosystem engineers can have far-reaching effects on the health of marine ecosystems [5,75,76]. We have shown for the first time that substantial waterborne transmission of SWD occurs in nature and probably plays a significant role in the spread of SWD. Only a small number of *L. zosterae* cells are needed to cause disease even at low water temperatures. Together, these findings demonstrate SWD is highly transmissible through the water column at low concentrations and has the potential to travel between spatially distinct populations over a wide range of temperatures.

## Data Availability

All data and statistical code used in this study is archived in the Dryad Data Repository: [77]. Supplementary material is available online [78].
